# Growth Differentiation Factor 15 as a Biomarker for Risk Stratification in the Cardiothoracic Surgery Intensive Care Unit

**DOI:** 10.3390/biom14121593

**Published:** 2024-12-13

**Authors:** Ricardo Ferreira, Tiago R. Velho, Rafael Maniés Pereira, Dora Pedroso, Beatriz Draiblate, Susana Constantino, Ângelo Nobre, Ana G. Almeida, Luís F. Moita, Fausto Pinto

**Affiliations:** 1 Department of Cardiothoracic Surgery, Hospital de Santa Maria, Unidade Local de Saúde de Santa Maria, 1649-028 Lisbon, Portugal; rmferreirast@gmail.com (R.F.); rafael.manies.pereira@gmail.com (R.M.P.); beatrizdraiblate@gmail.com (B.D.); angelolucasnobre@gmail.com (Â.N.); 2 Cardiothoracic Surgery Research Unit, Centro Cardiovascular da Universidade de Lisboa (CCUL@RISE), Faculdade de Medicina da Universidade de Lisboa, 1649-028 Lisbon, Portugal; 3 Innate Immunity and Inflammation Laboratory, Instituto Gulbenkian de Ciência, 2780-156 Oeiras, Portugal; dcpedroso@igc.gulbenkian.pt (D.P.); lferreiramoita@gmail.com (L.F.M.); 4 Department of Cardiopneumology, Escola Superior de Saúde da Cruz Vermelha Portuguesa, 1300-125 Lisbon, Portugal; 5 Angiogenesis Unit, Centro Cardiovascular da Universidade de Lisboa (CCUL@RISE), Faculdade de Medicina da Universidade de Lisboa, 1649-028 Lisbon, Portugal; sconstantino@medicina.ulisboa.pt; 6 Department of Cardiology, Hospital de Santa Maria, Unidade Local de Saúde de Santa Maria, 1649-028 Lisbon, Portugal; anagalmeida@gmail.com (A.G.A.); faustopin@gmail.com (F.P.); 7 Faculdade de Medicina da Universidade de Lisboa, 1649-028 Lisbon, Portugal

**Keywords:** GDF15, cardiac surgery, cardiopulmonary bypass, surgical aortic valve replacement, hemodynamic support, acute kidney injury

## Abstract

Growth Differentiation Factor 15 (GDF15) is an emerging biomarker that significantly increases during acute stress responses, such as infections, and is moderately elevated in chronic and inflammation-driven conditions. While evidence suggests that high levels of GDF15 in cardiac surgery are associated with worse outcomes, its utility as an evaluator of early postoperative complications remains unclear. This study aims to characterize the postoperative profile of GDF15 in patients undergoing isolated surgical aortic valve replacement, evaluating its association with short-term outcomes. Serum samples from patients undergoing cardiac surgery were collected preoperatively and at defined postoperative time points (1 h, 6 h, 12 h, 24 h, and 48 h) to measure GDF15 levels. GDF15 levels significantly increased after surgery, peaking at 6 h. A positive correlation was observed between GDF15 levels and both cardiopulmonary bypass and aortic cross-clamp times. Notably, patients who developed postoperative acute kidney injury (AKI) or required prolonged hemodynamic support had significantly higher GDF15 levels, with increased mechanical ventilation time and extended intensive care unit length of stay. Furthermore, GDF15 levels correlated with postoperative SOFA scores at 24 h after surgery. GDF15 may be a valuable biomarker for risk stratification and guiding therapeutic decisions in cardiac surgery patients. Higher GDF15 levels were significantly associated with prolonged hemodynamic support, postoperative AKI, and measures of illness severity.

## 1. Introduction

Growth Differentiation Factor 15 (GDF15), also known as serum macrophage inhibitory cytokine-1, is part of the TGFβ superfamily and plays a role in various physiological and pathological processes [1,2]. It is widely expressed in the liver, intestines, kidney, and placenta [3]. Under normal physiological conditions, circulating levels of GDF15 range from 0.1 to 1.2 ng/mL [3]. However, these concentrations can rise in response to cellular stress, as observed in cardiac and renal failure, chronic liver disease, and chronic inflammatory diseases [3].

Typically, GDF15 has very low expression in adult heart tissue but is significantly induced after injury [4]. Elevated plasma concentrations of GDF15 are independently associated with all-cause mortality and combined endpoints of death in both acute and chronic heart failure [5]. Additionally, increased levels of GDF15 have been reported following ischemic-reperfusion injuries or myocardial infarction, which are often linked to the use of extracorporeal circulation [6,7]. Despite its emergence recognition as a biomarker in cardiovascular diseases, the precise role of GDF15 in cardiac pathophysiology remains poorly understood.

In the context of cardiac surgery, elevated levels of GDF15 have been linked to postoperative atrial fibrillation and unfavorable outcomes, primarily due to myocardial and renal injury [8,9,10].

Despite these associations, the prognostic utility of GDF15 as a biomarker for predicting early outcomes following cardiac surgery is currently limited [11], with only a small amount of evidence available in the literature. Few studies have explored GDF15 levels and kinetics after surgery. Our study aims to characterize the postoperative profile of GDF15 following isolated surgical aortic valve replacement (SAVR) in the Cardiothoracic Surgery Intensive Care Unit (ICU) and to test its association with short-term postoperative outcomes.

## 2. Materials and Methods

### 2.1. Study Population

This prospective, observational, single-center study (part of the SVA-Inflammation Project) focuses on characterizing the biomarker profile following SAVR and its correlation with short-term outcomes in the ICU. Patients aged 18 and older proposed for SAVR were included in this study. Exclusion criteria were a history of previous cardiac surgery, concomitant procedures, history of neoplasia, immune disease, or ongoing medication with corticosteroids or immunosuppressants. The SAVR procedure was conducted according to the established protocols of our department, as previously described by our group [12].

Blood samples were systematically collected before surgery, and at 1 h, 6 h, 12 h, 24 h, and 48 h post-surgery. Plasma obtained from these samples was meticulously processed and stored at −80 °C. Clinical data collection included a thorough medical history detailing comorbidities and preoperative evaluations, specifications of the surgical procedure (including cardiopulmonary bypass and aortic cross-clamp times), and the postoperative course in the ICU and ward, followed by subsequent follow-up after discharge. The Sequential Organ Failure Assessment (SOFA) score was calculated 24 h after surgery in the ICU, as previously described [13].

All patients provided written informed consent, and the study was approved by the Institutional Ethics Committee (Comissão Ética CHLN, Ref.N.º23/18, 27 June 2023). The study was conducted in accordance with the Strengthening the Reporting of Observational Studies in Epidemiology (STROBE) guidelines.

### 2.2. Definitions or Outcomes Measures

We defined short-term outcomes as the occurrence of acute kidney injury (AKI), postoperative bleeding, use of hemodynamic support, and de novo atrial fibrillation (AF). AKI was assessed according to the Acute Kidney Injury Network (AKIN) criteria [12].

In the ICU, postoperative chest tube output was meticulously quantified on an hourly basis. Significant bleeding was defined as postoperative chest tube blood loss exceeding 600 mL within 12 h, in accordance with the criteria established by the International Initiative on Hemostasis Management in Cardiac Surgery [13,14].

Hemodynamic support was initiated for patients exhibiting persistent hypotension (mean arterial pressure < 65 mmHg), following adequate intravascular volume expansion with at least one of the following, according to patients’ characteristics and hemodynamics: (1) norepinephrine (dosage range 0.01–0.5 mcg/kg/min); (2) epinephrine (dosage range 0.01–0.05 mcg/kg/min); and (3) dobutamine (dosage range 5–20 mcg/kg/min). Patients requiring hemodynamic support for up to 24 h were categorized as needing “temporary support”, while those with sustained hemodynamic support for over 24 h were categorized as having “long-support”. None of the patients advanced to mechanical support.

De novo AF was diagnosed in patients without a preoperative history of AF, identified through electrocardiograms performed in the ICU.

### 2.3. GDF15 Measurement

GDF15 concentrations were determined using the human GDF15 ELISA kit (Quantikine ELISA kit, R&D Systems, Minneapolis, MN, USA), according to the manufacturer’s protocol and measured on a Tecan spectrophotometer plate-reader. The minimum detectable dose of human GDF15 in this kit ranges from 0.0 to 4.4 pg/mL (mean 2.0 pg/mL).

### 2.4. Statistical Analysis

Continuous variables are presented as median with interquartile range (IQR). The Mann–Whitney test was used for non-paired samples that did not meet the assumptions of normality and homoscedasticity. Categorical variables are reported in percentage or frequency and were analyzed using the chi-squared test.

To evaluate the association between GDF15 levels and CPB and aortic cross-clamp times, we used the Spearman’s rank-order correlation. Logistic regression was used to assess the association between GDF15 levels and other variables.

To assess differences in GDF15 levels, we performed multivariate linear regression and multiple linear mixed regression models. In these models, GDF15 levels were defined as the dependent variable, while the outcomes (e.g., long-support vs. temporary support) and time points were included as independent variables, along with their interaction.

All statistical tests conducted were two-sided, and *p*-values of ≤0.05 were considered statistically significant. Statistical analyses were performed using R, version 4.2.1 (R Foundation for Statistical Computing, Vienna, Austria).

## 3. Results

### 3.1. Patients’ Demographics

Sixty-two patients were included in this study. Baseline characteristics are detailed in Table 1. The median age of the patients was 73 (IQR 69–77). Nearly half (57.1%) were male, and hypertension was the most common comorbidity (82.5%). A significant proportion of patients also had dyslipidemia (61.9%) and diabetes mellitus (42.9%). The vast majority had preserved left ventricle function (92.1%). According to the New York Heart Association (NYHA) functional classification, almost all patients were symptomatic, with only 3.2% in NYHA Class I. The median EuroSCORE II was 1.27 (IQR 0.91–2).

Nearly one-third of the patients (19 patients—30.2%) had detectable preoperative GDF15 levels. These patients exhibited a higher incidence of diabetes mellitus, arterial hypertension, and peripheral arterial disease (Table 1), with a higher median EuroSCORE II. Moreover, patients with detectable preoperative levels of GDF15 had significantly lower body mass index and a lower prevalence of obesity compared to those with undetectable preoperative levels.

Postoperative details and complications are described in Table 2.

### 3.2. GDF15 Profile After Cardiac Surgery

**Figure 1:**
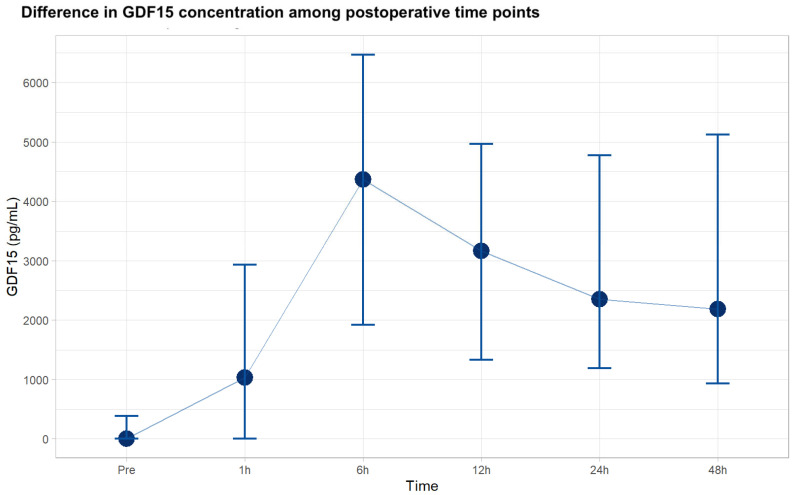
Differences in GDF15 concentration among postoperative time points. Values are presented with median and interquartile range for the 63 patients included, assessed by two replicates. GDF15: growth differentiation factor 15.

GDF15 values were detected preoperatively in 30.2% patients (19 patients). All patients presented detectable values postoperatively. Following surgery, GDF15 levels exhibited a notable increase, with a median value of 1030 pg/mL (IQR 0–2929) at 1 h after surgery. Levels peaked at 6 h, with a median of 4370 pg/mL (IQR 1925–6466), and then maintained relative stability up to 48 h (see Figure 1).

### 3.3. Cardiopulmonary Bypass, Aortic-Cross Clamp Times, and GDF15

We investigated whether GDF15 correlates with the postoperative systemic inflammatory response syndrome (SIRS) typically observed after CPB by assessing the association between GDF15 levels and CPB and aortic-cross clamp times. We found no strong correlation between CPB and aortic cross-clamp times and GDF15 values after surgery. However, we noted a positive correlation at 1h, 6h, and 12h post-surgery, with GDF15 levels increasing over these time points. Subsequently, a negative correlation was observed at 24 h and 48 h.

**Figure 2:**
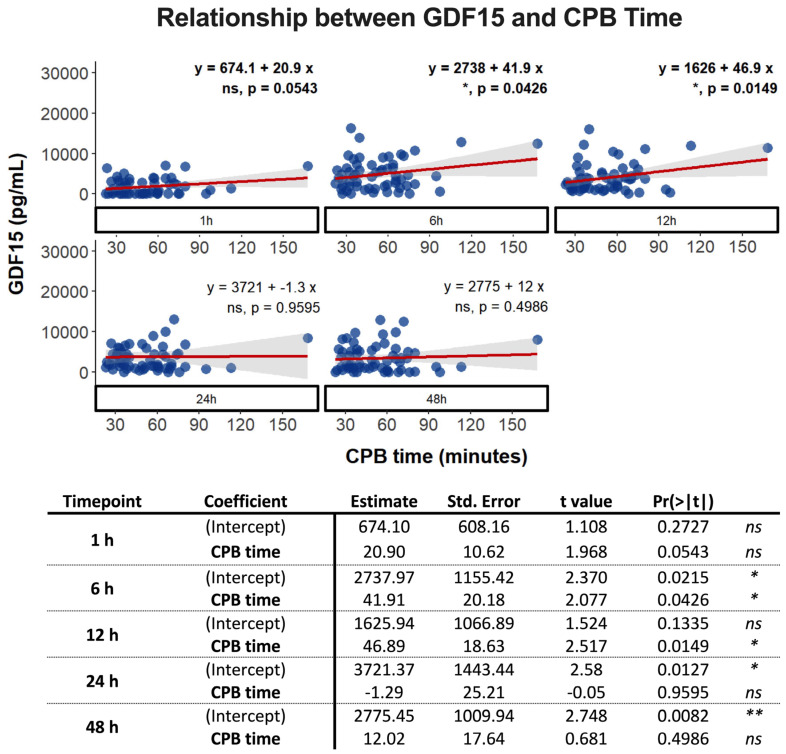
Multivariate linear regression analysis to assess the relationship between GDF15 levels (n = 63 with two replicates for each time-point: 1 h, 6 h, 12 h, 24 h, and 48 h) and cardiopulmonary (CPB) time. ns: non-significant; *: *p* < 0.05; **: *p* < 0.01.

While CPB time did not statistically explain GDF15 levels at 1 h after surgery (Figure 2), the *p*-value approached statistical significance. According to our model, every minute of CPB increased the mean GDF15 level by 20.9 pg/mL. The adjusted R-square value was approximately 0.05, indicating that only around 5% of the variation in GDF15 levels could be explained by CPB time at 1 h post-surgery.

At 6 h and 12 h post-surgery, the association between CPB time and GDF15 levels was statistically significant. The intercept and slope were 2738 pg/mL and 41.9 pg/mL for 6 h and 1626 pg/mL and 46.9 pg/mL for 12 h. The adjusted R-square values were 0.058 and 0.089, respectively. However, no statistically significant association was observed at 24 h and 48 h after surgery (Figure 2).

**Figure 3:**
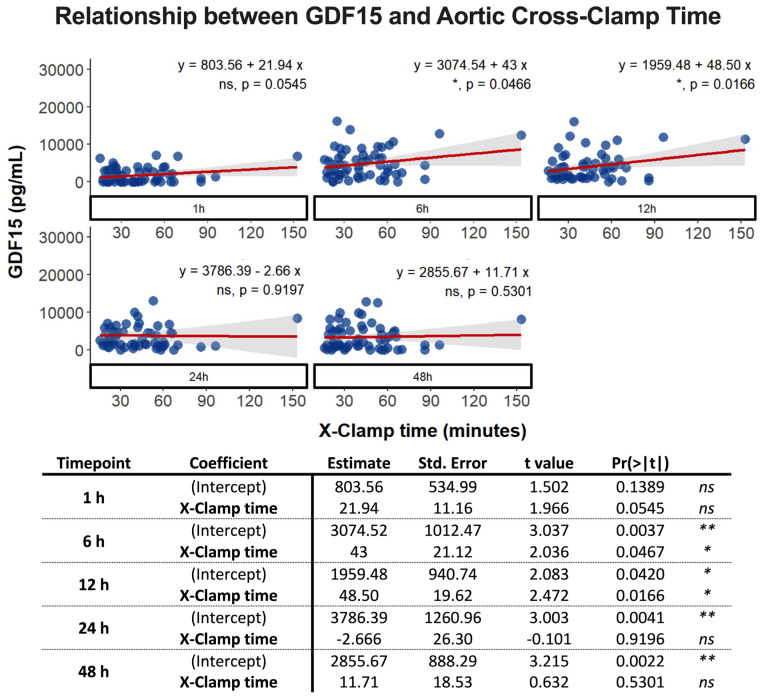
Multivariate linear regression analysis to assess the relationship between GDF15 levels (n = 63 with two replicates for each time-point: 1 h, 6 h, 12 h, 24 h, and 48 h) and aortic cross-clamp (X-clamp) time. ns: non-significant; *: *p* < 0.05; **: *p* < 0.01.

We observed a similar pattern when evaluating the association between aortic cross-clamp time and GDF15 levels. At 1 h after surgery, there was no statistically significant association between aortic cross-clamp time and GDF15 levels, although the *p*-value approached statistical significance (*p* = 0.0545). However, a statistically significant association was observed at 12 h (* *p* = 0.0466) and 24 h (* *p* = 0.0166) post-surgery (Figure 3). No statistically significant association was noted at 24 h and 48 h after surgery (Figure 3).

In our model, a linear association was evident for both CPB and aortic cross-clamp times at 1 h, 6 h, and 12 h; however, only the latter two showed statistically significant values. The adjusted R-squared values were not notably high, explaining up to 9% of the variation in GDF15 levels at 12 h. It is important to note that both variables only account for part of the observed variation, as numerous other factors impact GDF15 levels within such a complex system.

### 3.4. GDF15 and Postoperative Complications

Given that both CPB and aortic cross-clamp times are associated with heightened postoperative morbidity, including the need for inotropic or vasopressor support and postoperative organ dysfunction [15,16], we explored whether patients with postoperative complications displayed elevated levels of GDF15.

Indeed, we observed a linear association between GDF15 levels at 12 h and 24 h and postoperative SOFA score at 24 h. This observation suggests a connection between GDF15 and postoperative organ dysfunction.

**Figure 4:**
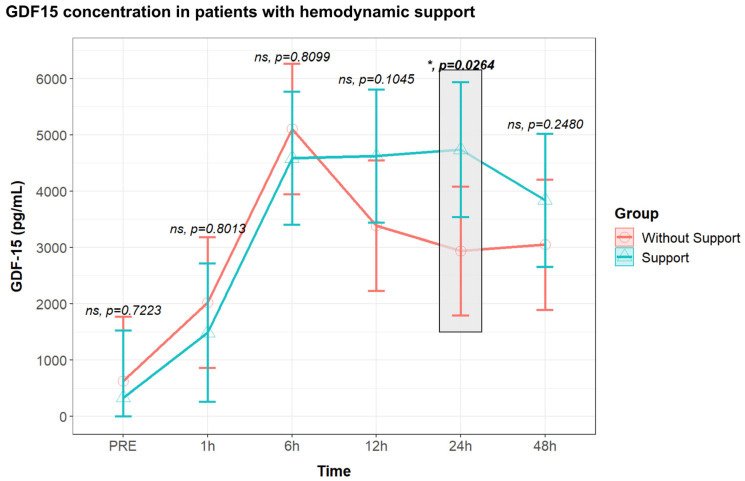
Postoperative GDF15 concentration in patients with (n = 31) and without (n = 32) hemodynamic support in the Intensive Care Unit. Values are presented with median and interquartile range, analyzed with a mixed linear regression, with fixed and random effects. All samples were assessed using two replicates. ns: non-significant; *: *p* < 0.05.

**Figure 5:**
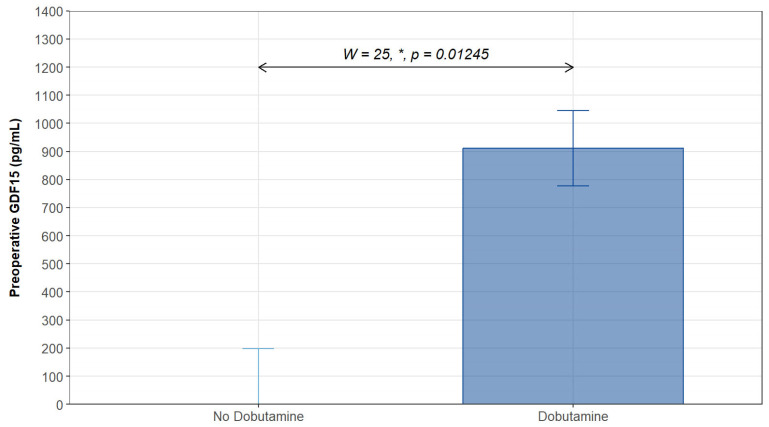
Median preoperative GDF15 concentration in patients who required postoperative dobutamine (n = 4), compared to those without (n = 15) any postoperative inotropic support. Only patients with detectable preoperative GDF15 levels (n = 19) were considered. Values are presented with median and interquartile range, analyzed with a Wilcoxon test, assessed by two replicates. *: *p* < 0.05.

However, we noted no differences in GDF15 levels between patients requiring or not requiring inotropic or vasopressor support at all time points (Figure 4). Interestingly, when we analyzed GDF15 levels based on the type of hemodynamic support (inotropic or vasopressor), we discovered that patients requiring postoperative inotropic support exhibited significantly higher preoperative levels of GDF15 (Figure 5).

Furthermore, we observed distinct GDF15 profiles between patients who experienced prolonged hemodynamic support (>24 h) during their ICU stay and those with only brief and temporary support.

**Figure 6:**
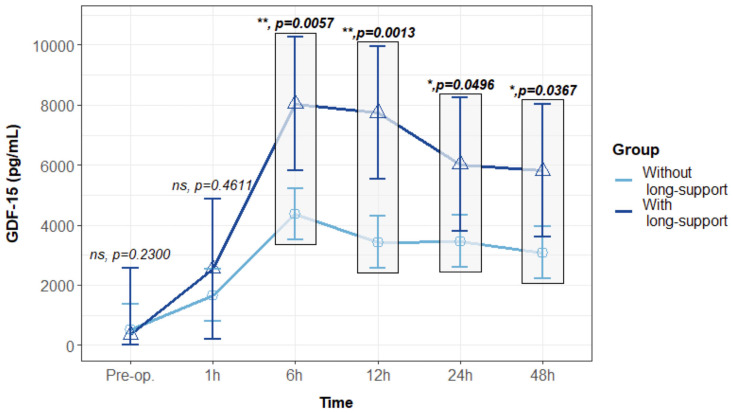
GDF15 postoperative profile comparing patients who required more than 24 h of hemodynamic support (n = 8) in the Intensive Care Unit (long-support), compared to those without hemodynamic support or just with a temporary necessity (n = 55). Values are presented with median and interquartile range, analyzed with a mixed linear regression, with fixed and random effects. Values were assessed using two replicates. ns: non-significant; *: *p* < 0.05; **: *p* < 0.01.

Patients requiring longer hemodynamic support, compared to those with only brief and temporary support, exhibited similar preoperative levels of GDF15 (516.9 ± 429.8 vs. 352.8 ± 1108.6 pg/mL, *p* = 0.891). Both groups demonstrated comparable values up to 1 h after surgery (Figure 6). However, at 6 h after surgery, patients requiring hemodynamic support for more than 24 h had significantly higher GDF15 levels (4365 ± 429.8 vs. 8040.9 ± 1108.6 pg/mL, ** *p* = 0.0057), a trend sustained at 12 h (3429.9 ± 429.9 vs. 7747.2 ± 1108.6 pg/mL, ** *p* = 0.0013), 24 h (3463.3 ± 429.8 vs. 6017.1 ± 1108.6 pg/mL, * *p* = 0.0496), and 48 h (3080.2 ± 429.9 vs. 5810.1 ± 1108.6 pg/mL, * *p* = 0.0367) after surgery (Figure 6). Interestingly, patients requiring longer hemodynamic support, with significantly higher GDF15 levels, experienced a significantly longer mechanical ventilation time (9 h IQR 4.8–13.2 vs. 5 IQR 3–6, ** *p* = 0.009), a higher postoperative SOFA score at 24 h (5.5 IQR 4–6.8 vs. 2 IQR 1–3, **** *p* < 0.0001), and a longer ICU length of stay (4 days IQR 3.3–5 vs. 2 days IQR 1–3, **** *p* < 0.0001).

**Figure 7:**
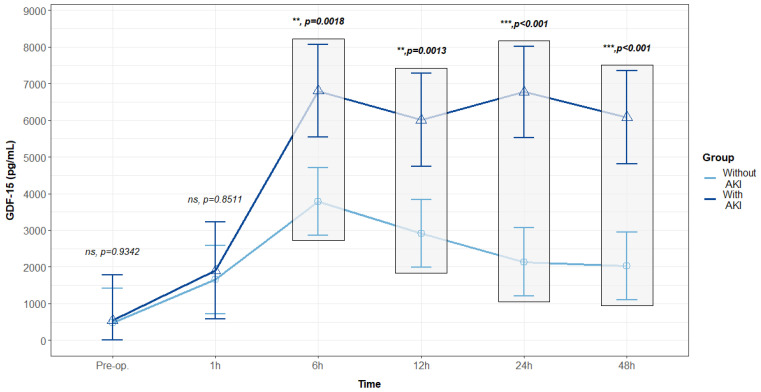
Median GDF15 concentrations comparing patients who developed postoperative acute kidney injury (n = 22) in the Intensive Care Unit with those without this complication (n = 41). Values are presented with median and interquartile range, analyzed with a mixed linear regression, with fixed and random effects. Values were assessed using two replicates. ns: non-significant; **: *p* < 0.01; ***: *p* < 0.001.

Similarly, we explored whether patients who developed postoperative AKI exhibited different levels of GDF15. Patients with postoperative AKI had similar preoperative levels of GDF15 and up to 1 h after surgery. However, from 6 h post-surgery, GDF15 levels significantly increased compared to patients without postoperative AKI. These elevated levels were sustained up to 48 h, while patients without AKI demonstrated a gradual decrease (Figure 7).

**Figure 8:**
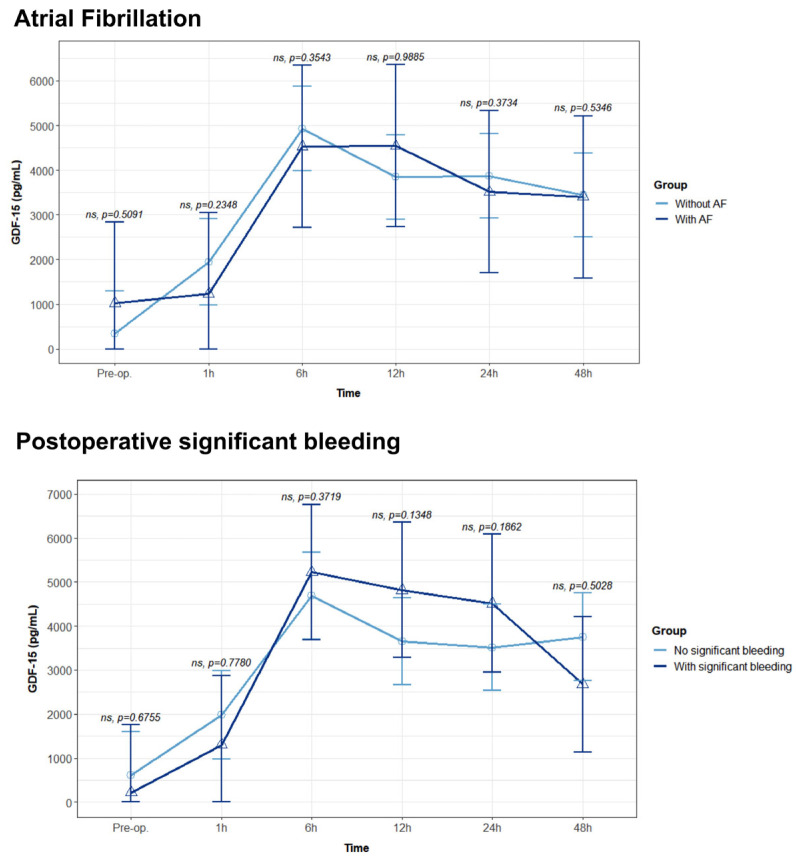
Median GDF15 concentration comparing patients with (n = 13) and without (n = 50) atrial fibrillation (AF) and with (n = 18) and without (n = 45) significant postoperative bleeding in the Intensive Care Unit. Values are presented with median and interquartile range, analyzed with a mixed linear regression, with fixed and random effects. Values were assessed using two replicates. ns: non-significant.

No differences were observed regarding GDF15 levels in patients who developed de novo AF and postoperative significant bleeding (Figure 8).

## 4. Discussion

This study aimed to characterize the profile of GDF15 in patients admitted to the Cardiothoracic Surgery ICU following surgical aortic valve replacement. Our findings revealed a significant increase in GDF15 levels after cardiac surgery with CPB, establishing a correlation between GDF15 levels and CPB and cross-clamping times. Additionally, we observed that patients who developed postoperative AKI or required hemodynamic support exhibited higher levels of GDF15. Previously, Kahli et al. conducted a small sampled study that described the kinetic increase in plasmatic GDF-15 levels during cardiac surgery with CPB [17]. However, our study provides a more comprehensive and clearly defined time course, spanning from preoperative to 48 h after surgery in the ICU, correlating with the occurrence of short-term outcomes.

The physiological role of GDF15 has garnered increased attention in recent years, with studies revealing its involvement in various conditions such as sepsis, obesity, cancer, and cachexia [18]. GDF15 is recognized as a stress response cytokine, with its expression heightened in response to diverse cellular stressors such as inflammation, hypoxia, tissue injuries, and myocardial ischemia [18]. In the context of cardiac surgery, cellular stress may elucidate the potential significance of characterizing GDF15 in this field. CPB has long been associated with the induction of a systemic inflammatory response syndrome (SIRS), which often leads to postoperative organ dysfunction. Previous research has established correlations between pro-inflammatory markers and postoperative organ dysfunction, with some target therapeutics [19] already in development [19,20].

Recent evidence suggests that postoperative organ dysfunction following cardiac surgery involves multiple factors beyond the inflammatory response induced by CPB alone. CPB appears to have a distinctive and specific metabolic signature of postoperative organ dysfunction [Velho et al., unpublished]. Therefore, it is crucial to identify new biomarkers capable of differentiating and evaluating the various pathways activated by CPB that lead to organ dysfunction. In this context, a new generation of biomarkers, such as GDF15, is being investigated to aid in predicting the severity of damage to vital organs associated with cardiac surgery [10,21,22]. The preoperative level of GDF15 serves as an independent marker of postoperative mortality and morbidity in cardiac surgery patients and those with heart failure, as it may reflect both severe cardiac disfunction and circulatory stress [23]. Our findings are consistent with this perspective, as patients requiring inotropic support exhibited significantly higher levels of GDF15, whereas those requiring only vasopressor support showed similar levels to those without such support. Furthermore, we observed that patients who required postoperative hemodynamic support and developed postoperative AKI presented higher postoperative levels of GDF15. Our finding is in line with previous studies that show that serum GDF15 levels are correlated with the estimated glomerular filtration rate [24].

Our study has indeed unveiled a correlation between GDF15 levels and both CPB and aortic cross-clamp times. Although we have only identified a modest correlation between CPB and aortic cross-clamp times and GDF15 levels, our data suggest a potential link between surgery duration and GDF15 release. It is widely recognized that CPB induces oxidative stress and triggers the release of inflammatory products [25,26]. Moreover, there is a suggestion that elevated levels of GDF15 may signify an increased demand for oxidative metabolism and tissue repair [27]. Some authors even propose that GDF15 could serve as a relevant measure of cardiopulmonary bypass function [23]. Consequently, elevated GDF15 levels in the context of cardiac surgery may reflect the metabolic processes associated with tissue repair and restoring homeostasis. This interpretation gains further support from the correlation we observed between GDF15 levels and the SOFA score in our study.

Given the direct, although modest, correlation between GDF15 and CPB duration, and the established link between CPB duration and postoperative organ dysfunction [15,16], our findings pave the way for further investigations into the role of GDF15 measurement as a modulator of interventions in the ICU. A recent study by Wollert et al. provides additional insight, demonstrating that patients presenting with acute myocardial infarction and elevated plasma GDF15 levels benefited more from an aggressive interventional approach compared to patients with lower levels of this cytokine [27]. This study highlights that a single measurement of GDF15 upon admission offers independent prognostic information on the risks of death and recurrent myocardial infarction, thereby improving the identification of patients who will benefit from an invasive strategy.

The early detection of GDF15 levels in the Cardiothoracic Surgery ICU following cardiac surgery with CPB holds promise for guiding treatment strategies and implementing timely aggressive measures to mitigate specific organ dysfunction. While the majority of studies have focused on the association between preoperative GDF15 levels and surgical outcomes, such as the occurrence of acute kidney injury associated with cardiac surgery [28], our study presents an association between perioperative GDF15 profiles in the ICU and short-term outcomes. In our investigation, patients with a prolonged need for hemodynamic support exhibited significantly higher GDF15 levels, requiring longer mechanical ventilation times and ICU length of stay (LOS). While further studies are needed to elucidate the precise role of GDF15 in this context, our study contributes by providing a descriptive postoperative time-course of GDF15 levels and correlating its kinetics with clinical outcomes such as AKI and the postoperative need for hemodynamic support.

Previous reports have linked GDF15 with the risk of major bleeding in various clinical scenarios, including patients with end-stage kidney disease, patients on hemodialysis [29], individuals with AF receiving anticoagulation therapy [30], and those with acute coronary syndrome undergoing antiplatelet treatment [31]. Although the exact mechanism remains unclear, GDF15 has been associated with the inhibition of platelet integrin activation [32] and has shown signs of causality with genetic effects on its levels and non-CABG-related bleeding [33]. However, our study did not observe differences in GDF15 levels between patients with significant postoperative bleeding and those without. Changes in aggregation and coagulation in the surgical setting, especially with the use of CPB, have unique specifications, and understanding the role of GDF15 in these cascades of events presents an opportunity for further research.

Moreover, based on our findings, it can be hypothesized that reducing CPB and aortic cross-clamping times, particularly by employing new surgical devices such as rapid deployment valves, could impact postoperative GDF15 levels. Our further projects aim to address this question.

In summary, our study reveals that patients undergoing cardiac surgery with CPB who develop postoperative AKI or require hemodynamic support exhibit significantly higher levels of GDF15, which in turn significantly influence the duration of mechanical ventilation and ICU LOS. This suggests that GDF15 may represent a novel biomarker for risk stratification and guiding therapeutic decisions in the context of cardiac surgery. This may represent an opportunity to provide a more personalized therapeutic approach, allowing proper resources allocation and patients’ stratification.

## 5. Conclusions

Our study has uncovered several significant findings concerning GDF15 levels in the context of cardiac surgery with CPB: (1) Following cardiac surgery with CPB, GDF15 levels significantly increase, peaking at 6 h post-surgery; (2) there is a statistically significant but modest correlation between both CPB and aortic cross clamp-times with GDF15 levels, suggesting a potential link between the duration of surgical procedures and the release of GDF15; (3) patients who develop postoperative AKI and require hemodynamic support exhibited elevated GDF15 levels, hinting at a possible association between GDF15 levels and postoperative complications; (4) GDF15 emerges as a promising biomarker for assessing the need for targeted approaches in the Cardiothoracic Surgery ICU, with potential implications for guiding therapeutic interventions. Further studies are necessary to thoroughly evaluate the potential of GDF15 as a biomarker to stratify patients who may benefit from more targeted interventions during the postoperative period.

## Figures and Tables

**Table 1:** Demographic details of the patients included in the study.

Variable	All Patients	Pre-op GDF15 Undetected	Pre-op Detected GDF15	*p*-Value	Long Support	Without Long Support	*p*-Value	AKI	No AKI	*p*-Value
N	63	44	19		8	55		22	41	
Age, years, median (IQR)	73 (69–77)	73 (67.5–75)	75 (69–78)	0.23	71.5 (64.8–78.5)	73 (69–77)	0.48	74 (65–78.3)	73 (69–76)	0.78
Male sex, n (%)	36 (57.1)	25 (56.8)	11 (57.9)	>0.99	5 (62.5)	31 (56.4)	>0.999	13 (59.1)	23 (56.1)	>0.999
BMI, kg/m^2^, mean ± SD	27.7 (25.2–29.7)	28.3 (26.6–30.4)	26 (24.1–27.7)	0.002	26.4 (23.4–30.6)	28 (25.8–29.7)	0.34	27.15 (24–28.4)	28.1 (26–30)	0.11
Hypertension, n (%)	52 (82.5)	34 (77.3)	19 (100)	0.03	7 (87.5)	45 (81.8)	>0.999	21 (95.5)	31 (75.6)	>0.999
Diabetes mellitus, n (%)	27 (42.9)	14 (31.8)	13 (68.4)	0.01	3 (37.5)	24 (43.6)	>0.999	9 (40.9)	18 (43.9)	>0.999
Dyslipidemia, n (%)	39 (61.9)	28 (66.6)	12 (63.2)	>0.99	4 (50)	35 (63.6)	0.47	13 (59.1)	26 (63.4)	0.79
Atrial fibrillation, n (%)	7 (11.1)	3 (6.8)	4 (21.1)	0.18	2 (25)	5 (9.1)	0.21	3 (13.6)	4 (9.8)	0.69
Chronic kidney disease, n (%)	3 (4.8)	1 (2.3)	2 (10.5)	0.21	1 (12.5)	2 (3.6)	0.34	3 (13.6)	0 (0)	0.04
Peripheral vascular disease, n (%)	5 (7.9)	1 (2.3)	4 (21.1)	0.03	0 (0)	5 (9.1)	>0.999	3 (13.6)	2 (4.9)	0.33
Cerebrovascular disease, n (%)	1 (1.6)	0 (0)	1 (5.3)	0.30	0 (0)	1 (1.8)	>0.999	0 (0)	1 (2.4)	>0.999
Chronic lung disease, n (%)	6 (9.5)	8 (18.2)	2 (10.5)	0.71	1 (12.5)	5 (9.1)	0.57	2 (9.1)	4 (9.8)	>0.999
Ischemic cardiopathy, n (%)	6 (9.5)	4 (9.1)	2 (10.5)	>0.99	2 (25)	4 (7.3)	0.16	0 (0)	6 (14.6)	0.08
Obesity, n (%)	14 (22.2)	13 (29.5)	1 (5.3)	0.047	2 (25)	12 (21.8)	>0.999	4 (18.2)	10 (24.4)	0.75
Previous cardiac surgery, n (%)	0 (0)	0 (0)	0 (0)	>0.99	0 (0)	0 (0)	---	0 (0)	0 (0)	---
LV function, n (%) Preserved Moderate 31–50 Poor LV function (21–30%) Very Poor LV function (<20%)	58 (92.1) 2 (3.2) 2 (3.2) 1 (1.6)	42 (95.5) 0 (0) 2 (4.5) 0 (0)	16 (84.2) 2 (10.5) 0 (0) 1 (5.3)	0.16 0.09 >0.99 0.30	6 (75) 1 (12.5) 0 (0) 1 (12.5)	53 (96.4) 0 (0) 2 (3.6) 0 (0)	0.02 0.08 0.58 0.08	19 (86.4) 1 (4.5) 1 (4.5) 1 (4.5)	39 (95.1) 1 (2.4) 1 (2.4) 0 (0)	0.22 0.65 0.65 0.17
NYHA, n (%) I II III IV	2 (3.2) 18 (28.6) 42 (66.7) 1 (1.6)	2 (4.5) 7 (15.9) 32 (72.7) 1 (2.3)	0 (0) 6 (31.6) 13 (68.4) 0 (0)	>0.99 0.19 0.77 >0.99	0 (0) 8 (100) 0 (0) 0 (0)	2 (3.6) 18 (32.7) 34 (61.8) 1 (1.8)	0.58 0.01 0.001 0.70	0 (0) 8 (36.4) 14 (63.6) 0 (0)	2 (4.9) 10 (24.4) 28 (68.3) 1 (2.4)	0.29 0.32 0.71 0.46
EuroSCORE II (IQR)	1.27 (0.91–2)	1.09 (0.9–1.53)	1.95 (1.16–2.34)	0.04	1.43 (0.91–2)	1.26 (0.92–2.16)	0.90	1.74 (0.95–2.6)	1.23 (0.9–1.8)	0.27

EuroSCORE II: European System for Cardiac Operative Risk Evaluation II; BMI: body mass index; SD: standard deviation; IQR: interquartile range; LV: left ventricle; NYHA: New York Heart Association.

**Table 2:** Perioperative details.

Variable	All Patients	Pre-op GDF15 Undetected	Pre-op Detected GDF15	*p*-Value	Long Support	Without Long Support	*p*-Value	AKI	No AKI	*p*-Value
N	63	44	19		8	55		22	41	
CPB time, min, median (IQR)	50 (34–66)	50 (36–66)	49 (33–69)	0.82	60.5 (37.8–104.8)	49 (34–66)	0.18	57.5 (37.8–67.5)	47 (33–66)	0.15
Aortic cross-clamp, min, median (IQR)	40 (26–56)	39.5 (26–55.8)	42 (25–56)	0.76	49 (25.5–88)	39 (26–55)	0.28	42 (29.3–54.5)	38 (22.5–57)	0.29
Hemodynamic support, n (%)	31 (49.2)	21 (47.7)	10 (52.6)	0.79	---	---	---	14 (63.6)	17 (41.5)	0.12
Mechanical ventilation, hour, median (IQR)	5 (3.8–7)	5 (4–7)	5 (3–9)	0.75	9 (4.8–13.2)	5 (3–6)	0.009	5 (3.8–8.3)	5 (3.3–6)	0.41
Post-op bleeding 24 h, mL, median (IQR)	400 (300–600)	400 (300–600)	300 (300–600)	0.33	600 (300–700)	400 (300–600)	0.24	400 (300–550)	400 (300–700)	0.95
Re-exploration due to bleeding, n (%)	1 (1.6)	1 (2.3)	0 (0)	>0.99	0 (0)	1 (1.8)	>0.99	0 (0)	1 (2.4)	>0.99
De novo AF, n (%)	13 (20.6)	8 (18.2)	5 (26.3)	0.51	1 (12.5)	12 (21.8)	>0.99	4 (18.2)	9 (40.9)	>0.99
AKI, n (%)	22 (34.9)	12 (0.27)	10 (52.6)	0.08	6 (75)	16 (29.1)		---	---	---
Stroke, n (%)	1 (1.6)	1 (2.3)	0 (0)	>0.99	0 (0)	1 (1.8)	>0.99	0 (0)	1 (2.4)	>0.99
SOFA score, median (IQR)	2 (1–4)	2 (1–3.75)	2 (1–4)	0.95	5.5 (4–6.8)	2 (1–3)	0.009	5 (3.8–8.3)	5 (3.3–6)	0.41
Death, n (%)	0 (0)	0 (0)	0 (0)	---	0 (0)	0 (0)	---	0 (0)	0 (0)	---
ICU LOS, days, median (IQR)	2 (2–3)	2 (1–3)	2 (2–3.3)	0.62	4 (3.3–5)	2 (1–3)	<0.0001	3 (2–5)	2 (1.5–3)	0.15

AF: Atrial fibrillation; AKI: acute kidney injury; CPB: cardiopulmonary bypass; ICU: intensive care unit; IQR: interquartile range; LOS: length of stay; SOFA: sequential organ failure assessment score.

## Data Availability

The derived data generated in this research will be shared upon reasonable request to the corresponding author.
